# Supplementary material for: The endocannabinoid anandamide is an airway relaxant in health and disease
Source: Nat Commun. 2022 Nov 17;13:6941. doi: 10.1038/s41467-022-34327-0 (PMC9672354; doi:10.1038/s41467-022-34327-0)
Supplement: Supplementary file 1 — Supplementary Information [file 41467_2022_34327_MOESM1_ESM.pdf]

Supplementary Information for

The endocannabinoid anandamide is an airway relaxant in health and disease

Anandamide/FAAH pathway mediates airway relaxation

Annika Simon[1], Thomas von Einem[2], Alexander Seidinger[1], Michaela Matthey[1], Laura Bindila[3], *Daniela Wenzel[1,2]

Correspondence to: daniela.wenzel@rub.de

This pdf file includes:

Supplementary Figures 1 to 6

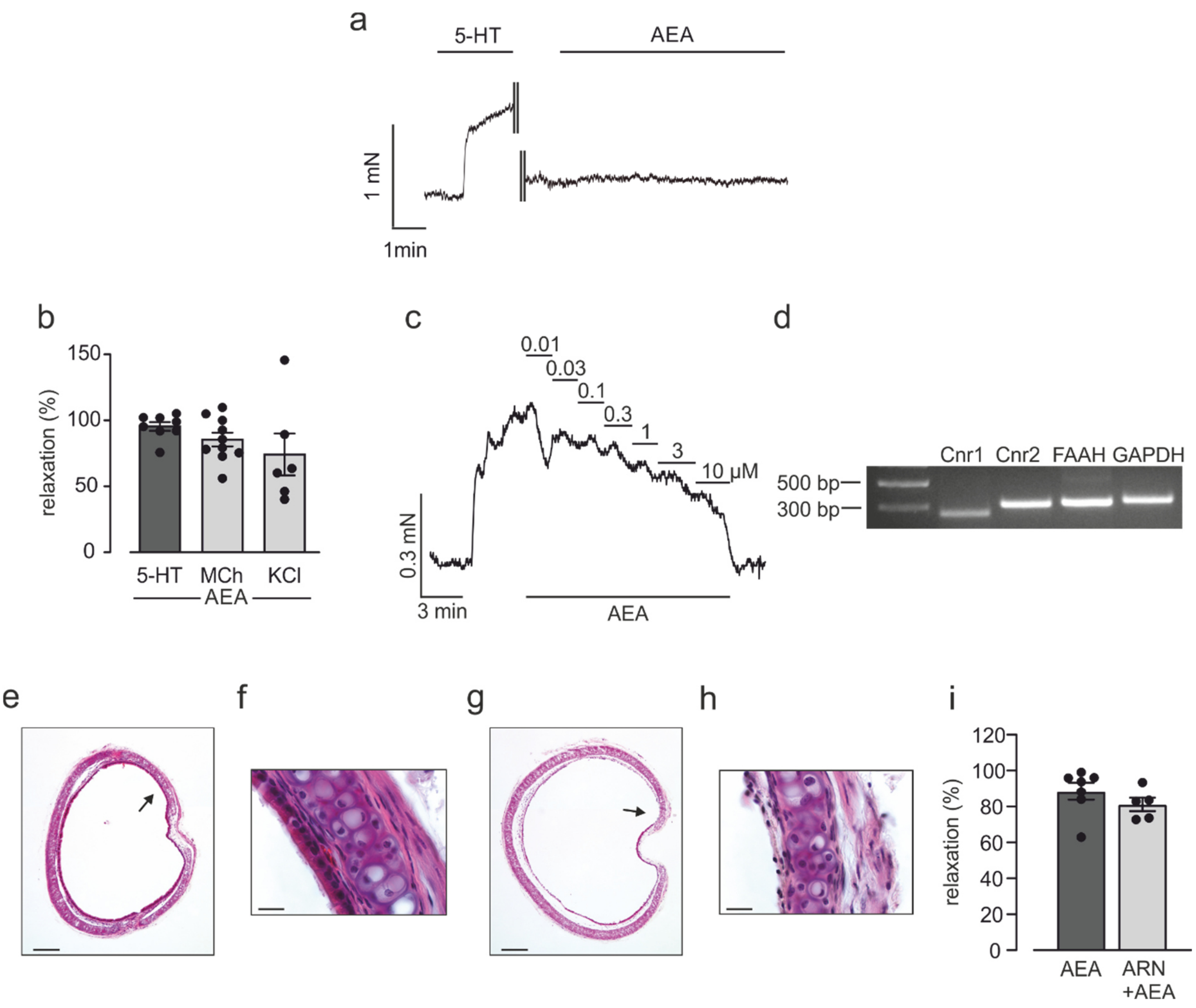

Supplementary Figure 1. Anandamide (AEA) induces airway relaxation after pre-constriction with different agonists, the effect is partly epithelium-dependent. a) Original trace of isometric force measurements in a myograph shows that AEA (10 µM) does not affect baseline tone of tracheal rings; airway constriction by serotonin (5-HT, 10 µM) confirms that tone regulation is intact. b) Statistical analysis of airway relaxation in C57BL/6J mouse trachea by AEA (10 µM) after submaximal pre-constriction with 5-HT (3 µM, *n*=8), MCh (0.1 µM, *n*=10) and KCl (28 mM, *n*=6) shows comparable effects irrespective of the pre-constrictor. Results were derived from measurements of independent samples. c) Original trace of isometric force measurements demonstrates dose-dependent airway relaxation by AEA in C57BL/6J mouse trachea. d) PCR analysis of C57BL/6J mouse tracheas illustrates low CB1 but stronger CB2 and FAAH expression, note that 35 cycles were used for Cnr1, Cnr2 and FAAH while 25 cycles

were applied for GAPDH. Analysis was performed twice. e-h) Hematoxylin and eosin (H&E) staining demonstrates intact epithelium in native C57BL/6J mouse trachea (e,f) while the epithelium is absent after mechanical denudation (g,h), scale bar = 20 µm (f,h) and scale bar = 200 µm (e,g). (f and h are taken from tracheal regions indicated with arrows in e and g). Staining was performed twice. i) Statistical analysis of airway relaxation by AEA (*n*=7 independent samples) reveals that this is independent from N-acylethanolamine acid amide hydrolase (NAAA) that can be inhibited by ARN726 (10 µM, *n*=5 independent samples). All data are presented as mean values ± SEM. Source data are provided as a Source Data file.

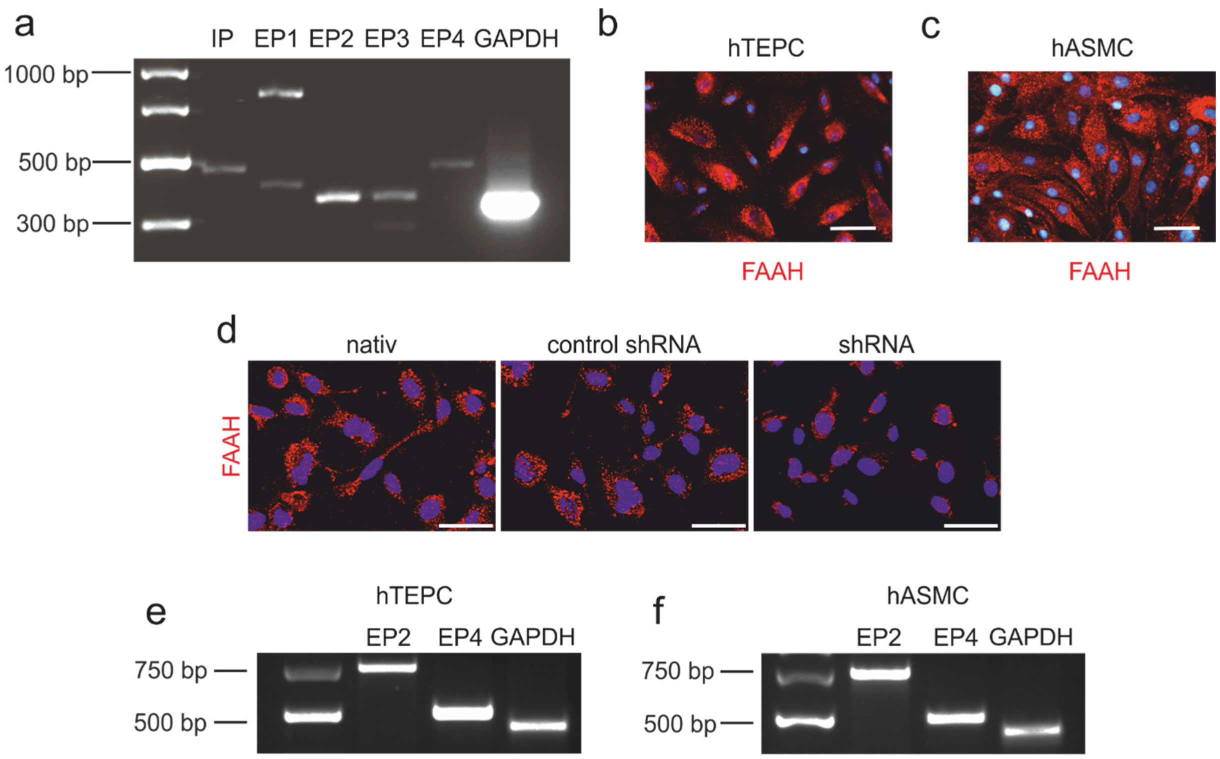

Supplementary Figure 2. Prostaglandin receptors and fatty acid amide hydrolase (FAAH) are expressed in mouse trachea and/or in human epithelial (hTEPC) as well as human smooth muscle cells (hASMC). a) PCR analysis demonstrates expression of prostacyclin receptor (IP) and PGE2 receptors EP1, EP2, EP3 and EP4. Note: band at 830 bp is protein kinase N1 (Pkn1) that shows sequence homology with EP1, for EP3 2 transcript variants are detected. PCR was performed twice. b,c) Immunostaining reveals FAAH expression in hTEPC (b) and hASMC (c) (red = FAAH), scale bar = 50 µm. Staining was performed 3 times. d) Immunostaining in human umbilical vein endothelial cells (HUVEC) demonstrates specificity of FAAH antibody staining (red) by knockdown using FAAH shRNA, scale bar = 50 µm. Staining was performed twice. e,f) PCR analysis shows EP2 and EP4 expression in hTEPC (e) and hASMC (f). PCR was performed twice.

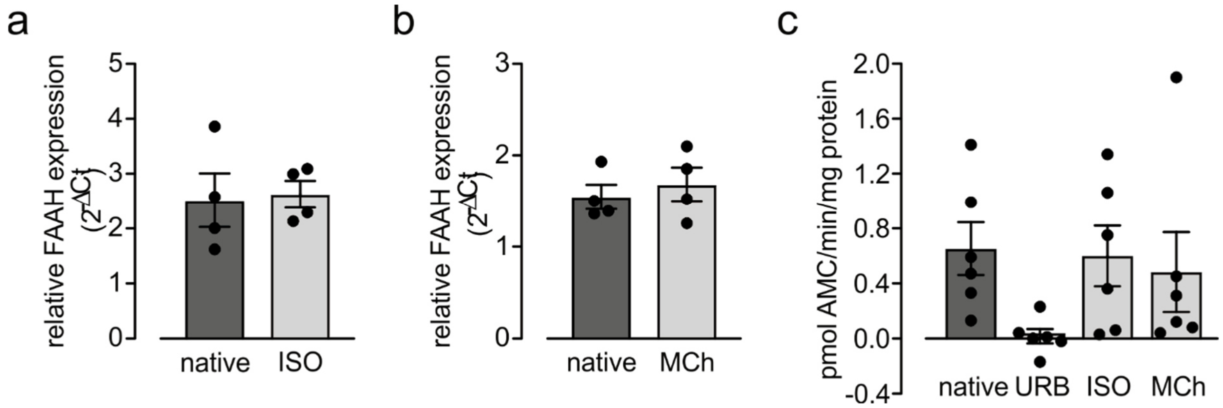

Supplementary Figure 3. FAAH expression and activity are independent from beta adrenergic or cholinergic signaling. a,b) QPCR analysis reveals that FAAH expression is unaltered by isoprenaline (ISO, 10 μM, a) and methacholine (MCh, 10 μM, b) incubation (*n*=4 independent C57BL/6J mice). c) Statistical analysis demonstrates that FAAH activity is independent from ISO and MCh (10 μM each) treatment (*n*=6 independent C57BL/6J mice); URB597 (URB, 10 μM). 7-amino-4-methylcoumarin (AMC). All data are presented as mean values ± SEM. Source data are provided as a Source Data file.

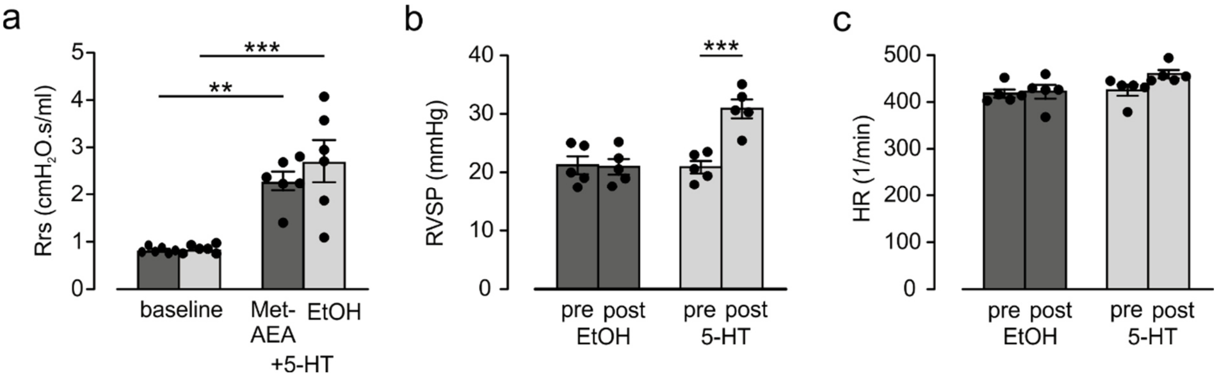

Supplementary Figure 4. Inhalation of solvent EtOH does not affect right ventricular systolic pressure (RVSP) and heart rate (HR). a) Analysis of airway resistance (Rrs) at baseline and after the subsequent inhalation of 25 mg/ml 5-HT together with Met-AEA (0.5 mg per mouse) or the solvent EtOH in Balb/c mice with acute OVA asthma. Met-AEA cannot prevent the increase of airway resistance by 5-HT (*n*=6 independent animals). Repeated measures two way ANOVA, baseline vs Met-AEA **p=0.002 and baseline vs EtOH ***p=$3*10^{-4}$. b) Analysis of right ventricular systolic pressure (RVSP) before (pre) and after (post) inhalation of the solvent EtOH or 5-HT (50 mg/ml per mouse); AEA inhalation has no effect on RVSP (*n*=5 independent C57BL/6J mice). Repeated measures two way ANOVA, pre vs post 5-HT ***p=$5.1*10^{-4}$. c) Analysis of heart rate (HR) before (pre) and after (post) inhalation of the solvent EtOH or 5-HT (50 mg/ml per mouse). EtOH and 5-HT have no effect on HR. (*n*=5 independent C57BL/6J mice). All data are presented as mean values ± SEM. Source data are provided as a Source Data file.

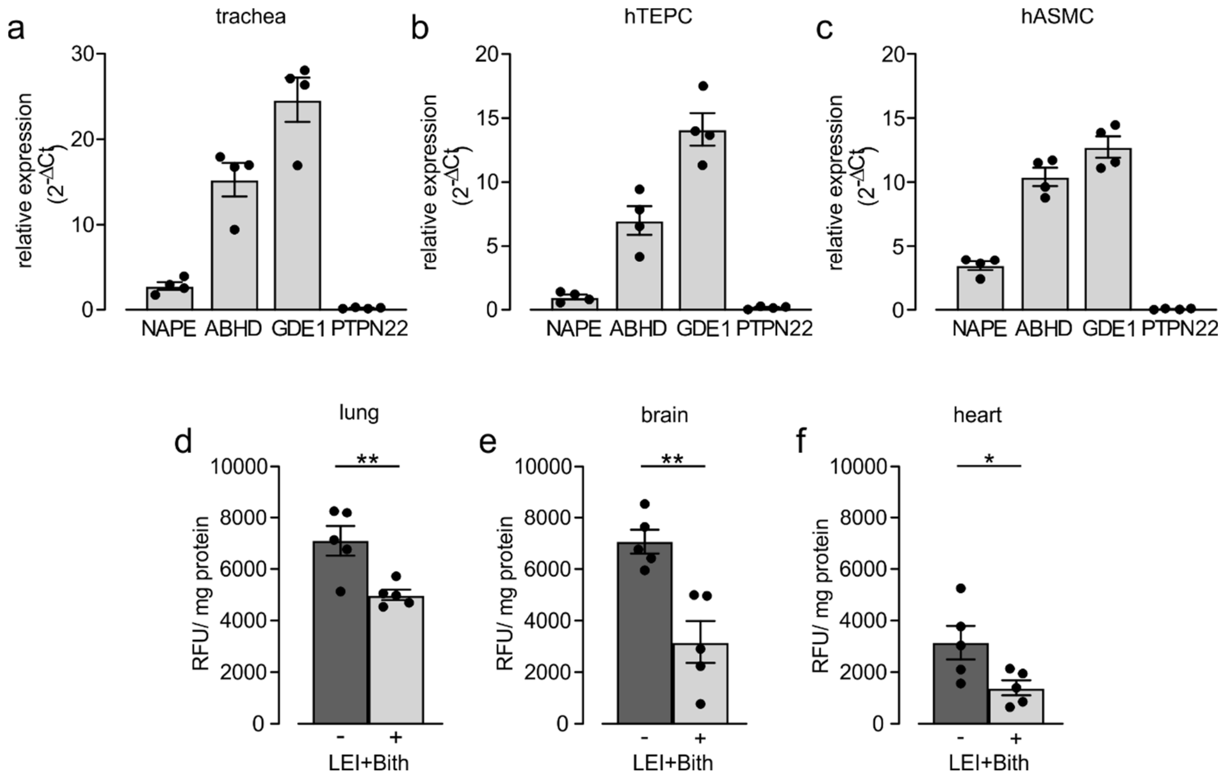

Supplementary Figure 5. Enzymes that synthesize AEA are expressed in different mouse organs and human airway cells. a-c) QPCR analysis reveals expression of N-acyl-phosphatidyl-ethanolamine phospholipase D (NAPE), a/b-hydrolase domain-containing protein (ABDH), glycerophosphodiesterase 1 (GDE1) and protein tyrosine phosphatase nonreceptor type 22 (PTPN22) in C57BL/6J mouse trachea (a), hTEPC (b) and hASMC (c) (*n*=4 independent animals/samples). d-f) NAPE-PLD activity can be detected in C57BL/6J mouse lung (d), brain (e) and heart (f), (*n*=5 independent C57BL/6J mice). Unpaired two-tailed student`s *t*-test (lung ***p*=0.0087, brain ***p*=0.0032, heart **p*=0.0397); LEI-410 (LEI, 33 µM), bithionol (Bith, 15 µM), relative fluorescence units (RFU). All data are presented as mean values ± SEM. Source data are provided as a Source Data file.

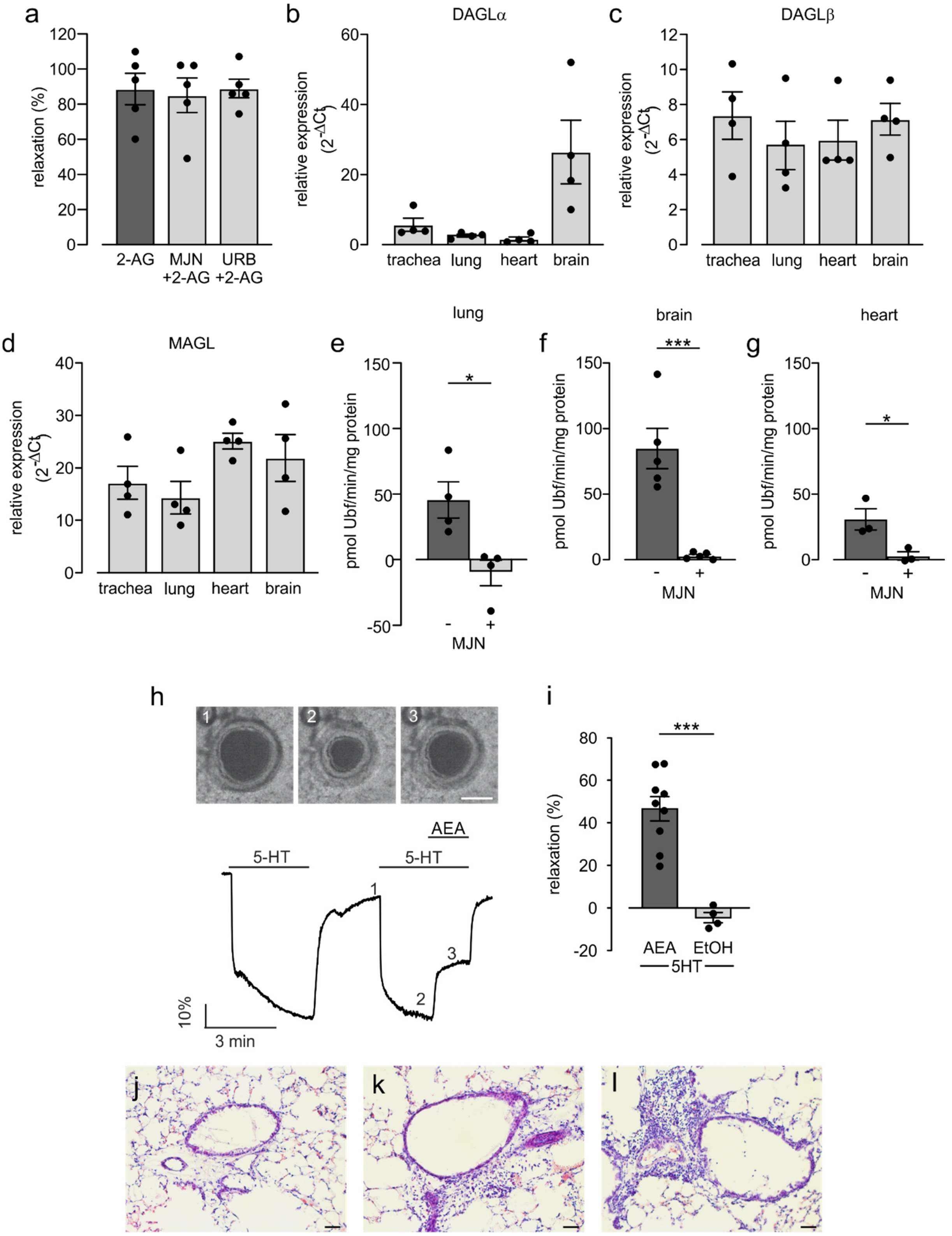

Supplementary Figure 6. 2-Arachidonlyglycerol (2-AG)-induced airway relaxation is independent from MAGL and FAAH, AEA also induces airway relaxation in healthy Balb/c mice. a) Statistical analysis of airway tone in C57BL/6J mouse trachea in response to 2-AG indicates that 2-AG evokes airway relaxation independent from monoacylglycerol lipase

(MAGL) and FAAH (*n=5* independent samples); MJN110 (MJN, 10 µM), URB (URB597, 10 µM). b-d) QPCR experiments show expression of diacylglycerol lipase (DAGL)α (b), DAGLβ (c) and MAGL (d) in C57BL/6J mouse trachea (*n=4*), lung (*n=4*), heart (*n=4*) or brain (*n=4*), all independent animals. e-g) Analysis demonstrates MAGL activity in C57BL/6J mouse lung (*n*=4) (e), brain (*n*=5) (f) and heart (*n*=3) (g), all independent animals, umbelliferone (Ubf). Unpaired two-tailed student`s *t*-test, lung $*p=0.017$, brain $***p=7*10^{-4}$, heart $*p=0.033$. h) Top: Phase contrast microscopy pictures of a small intrapulmonary mouse airway in healthy Balb/c mouse. 1-3) Pictures represent time points during perfusion in the graph (bottom), scale bar = 50 µm. Bottom: Original trace of changes in airway lumen area. AEA (10 µM) completely reverses the reduction of lumen area by 5-HT (0.1 µM). i) Statistical analysis reveals strong airway relaxation by AEA in intrapulmonary airways of healthy Balb/c mice (AEA *n*=9, EtOH *n*=4 independent animals). Unpaired two-tailed student`s *t*-test $***p=1.3*10^{-4}$. j-l) H&E staining demonstrates no inflammatory cells in controls (j) and invasion of inflammatory cells around the airways of precision-cut lung slices confirming the successful induction of OVA-dependent acute (k) and chronic (l) asthma, scale bar = 20 µm. Staining was performed twice. All data are presented as mean values ± SEM. Source data are provided as a Source Data file.
