# Supplementary material for: The endocannabinoid anandamide is an airway relaxant in health and disease
Source: Nat Commun. 2022 Nov 17;13:6941. doi: 10.1038/s41467-022-34327-0 (PMC9672354; doi:10.1038/s41467-022-34327-0)
Supplement: Supplementary file 3 — Reporting Summary [file 41467_2022_34327_MOESM3_ESM.pdf]

nature portfolio

Corresponding author(s): Wenzel, Daniela

Last updated by author(s): 22-09-07

Reporting Summary

Nature Portfolio wishes to improve the reproducibility of the work that we publish. This form provides structure for consistency and transparency in reporting. For further information on Nature Portfolio policies, see our Editorial Policies and the Editorial Policy Checklist.

Statistics

For all statistical analyses, confirm that the following items are present in the figure legend, table legend, main text, or Methods section.

n/a	Confirmed	
☐	☒	The exact sample size (n) for each experimental group/condition, given as a discrete number and unit of measurement
☐	☒	A statement on whether measurements were taken from distinct samples or whether the same sample was measured repeatedly
☐	☒	The statistical test(s) used AND whether they are one- or two-sided<br>*Only common tests should be described solely by name; describe more complex techniques in the Methods section.*
☒	☐	A description of all covariates tested
☐	☒	A description of any assumptions or corrections, such as tests of normality and adjustment for multiple comparisons
☐	☒	A full description of the statistical parameters including central tendency (e.g. means) or other basic estimates (e.g. regression coefficient) AND variation (e.g. standard deviation) or associated estimates of uncertainty (e.g. confidence intervals)
☐	☒	For null hypothesis testing, the test statistic (e.g. F, t, r) with confidence intervals, effect sizes, degrees of freedom and P value noted<br>*Give P values as exact values whenever suitable.*
☒	☐	For Bayesian analysis, information on the choice of priors and Markov chain Monte Carlo settings
☒	☐	For hierarchical and complex designs, identification of the appropriate level for tests and full reporting of outcomes
☒	☐	Estimates of effect sizes (e.g. Cohen's d, Pearson's r), indicating how they were calculated

Our web collection on statistics for biologists contains articles on many of the points above.

Software and code

Policy information about availability of computer code

Data collection	Isometric force measurements were performed with the LabChart8 software (ADInstruments, Oxford, UK) connected to the Multi Myograph System 620 (DMT, Aarhus, Denmark), precision cut lung slices were imaged with the help of Lumen Calc 2.4, National Instruments, Texas, USA. Histological sections were imaged using the AxioVision Rel. 4.8 software (Carl Zeiss, Germany). Fluorescence assays were analyzed using the i-control software provided with the Tecan Infinite M1000 Pro reader (Tecan GmbH, Austria). QPCR was performed with the CFX96 Touch™ Real-Time PCR Detection System (Bio-Rad Laboratories GmbH, Hercules, CA, USA). Airway resistance was recorded using a flexiVent system (Scireq, Canada). Right ventricular catheter measurements were performed with a Millar Aria 1 system (Millar, USA) together with the LabChart8 software.
Data analysis	Isometric force measurements and catheter experiments were analyzed with the LabChart8 software. Airway resistance was determied using the flexiware 8 software (Scireq). Statistical analysis was performed using Excel 365 and GraphPad Prism 5.0 (GraphPad Software, San Diego, USA). Figures were generated with Corel Draw 18 (Corel Corporation, Ottawa, Canada).

For manuscripts utilizing custom algorithms or software that are central to the research but not yet described in published literature, software must be made available to editors and reviewers. We strongly encourage code deposition in a community repository (e.g. GitHub). See the Nature Portfolio guidelines for submitting code & software for further information.

March 2021

Data

Policy information about availability of data

All manuscripts must include a data availability statement. This statement should provide the following information, where applicable:

- Accession codes, unique identifiers, or web links for publicly available datasets
- A description of any restrictions on data availability
- For clinical datasets or third party data, please ensure that the statement adheres to our policy

All data associated with this study are present in the paper or the supplementary materials. Additional data related to this paper are provided in the source data file.

Field-specific reporting

Please select the one below that is the best fit for your research. If you are not sure, read the appropriate sections before making your selection.

☒ Life sciences ☐ Behavioural & social sciences ☐ Ecological, evolutionary & environmental sciences

For a reference copy of the document with all sections, see nature.com/documents/nr-reporting-summary-flat.pdf

Life sciences study design

All studies must disclose on these points even when the disclosure is negative.

Sample size	For in vivo experiments sample sizes were calculated by biometric analysis (power of 0.8). For ex vivo experiments sample sizes were chosen depending on the method applied. For LC-MRM analysis n=10 was measured in the same run. For all other experiments n= 3-9 was applied. According to our experience with these methods this is sufficient for characterizing the functional differences.
Data exclusions	In isometric force measurements only measurements with pre-contraction forces of at least 0.2 mN were used for statistics.
Replication	For qPCR technical triplicates were used. Qualitative PCRs and immunostainings were performed at least twice. The number of independent experiments is indicated in the legends of the figures. Replications in some myograph experiments failed on a few occasions due to problems with the pH of the measurement solution (human error). These experiments were not included into the manuscript.
Randomization	Mice, tissues and cell samples were all randomly assigned to the experimental groups.
Blinding	Invetigators were not blinded because data points were recorded by the software and analysis was performed at specific time points after drug application

Reporting for specific materials, systems and methods

We require information from authors about some types of materials, experimental systems and methods used in many studies. Here, indicate whether each material, system or method listed is relevant to your study. If you are not sure if a list item applies to your research, read the appropriate section before selecting a response.

Materials & experimental systems

n/a	Involved in the study	
☐	☒	Antibodies
☐	☒	Eukaryotic cell lines
☒	☐	Palaeontology and archaeology
☐	☒	Animals and other organisms
☒	☐	Human research participants
☒	☐	Clinical data
☒	☐	Dual use research of concern

Methods

n/a	Involved in the study	
☒	☐	ChIP-seq
☒	☐	Flow cytometry
☒	☐	MRI-based neuroimaging

Antibodies

Antibodies used	FAAH polyclonal antibody (Cayman Chemical, Item No 101600, 1:50-1:100), anti-asmac antibody, mouse, polyclonal (clone1A4, Sigma-Aldrich, A5228, 1:800), secondary antibodies conjugated with Cy3 and Cy5 (1:400, 711-175-152, Jackson ImmunoResearch Laboratories, USA) were applied
Validation	anti-FAAH: reduced staining after shRNA knockdown of FAAH in HUVEC (this study), maunfacturer`s website: Immunohistochemistry analysis of formalin-fixed, paraffin-embedded (FFPE) human brain tissue after heat induced antigen retrieval in pH 6.0 citrate buffer. After incubation with Fatty Acid Amide Hydrolase Polyclonal Antibody (Item No. 101600) at a 1:40 dilution, slides were incubated with biotinylated secondary antibody, followed by alkaline phosphatase-streptavidin and chromogen (DAB).

anti-asmac: manufacturer`s website: IHC signal in vessels of human appendix, IF signal in Rat2 cells

Eukaryotic cell lines

Policy information about cell lines

Cell line source(s)	human tracheal epithelial cells (hTEPC, Provitro AG, Berlin, Germany), human airway smooth muscle cells (hASMC, Provitro AG), human umbilical vein endothelial cells (HUVEC, Provitro AG)
Authentication	hASMC were tested in Matthey et al. Sci Transl Med 2017 in [Ca2+]i imaging experiments, hTEPC and HUVEC were not tested.
Mycoplasma contamination	Cells were kept separate from primary cell culture cells where mycoplasma contamination could occur (separate cell culture room and liquid nitrogen tank) . Cells were not tested for mycoplasma contamination.
Commonly misidentified lines (See ICLAC register)	none

Animals and other organisms

Policy information about studies involving animals; ARRIVE guidelines recommended for reporting animal research

Laboratory animals	For experiments 8-12 week old female C57BL/6J (Charles River) and Balb/c mice (Janvier) were used, FAAH-/- and CB1/2-/- mice were kindly provided by the Institute of Molecular Psychiatry of the University Hospital Bonn. All mice were housed in standard cages on a 12h light-dark cycle at 21°C and 50% humidity and had ad libitum access to food and water.
Wild animals	none
Field-collected samples	none
Ethics oversight	Animal experiments were performed in compliance with the guidelines of the German law and were approved by the LANUV (Landesamt für Natur, Umwelt und Verbraucherschutz NRW, Germany).

Note that full information on the approval of the study protocol must also be provided in the manuscript.
